# Pre-sowing Seed Treatments in Direct-seeded Early Rice: Consequences for Emergence, Seedling Growth and Associated Metabolic Events under Chilling Stress

**DOI:** 10.1038/srep19637

**Published:** 2016-01-19

**Authors:** Weiqin Wang, Qian Chen, Saddam Hussain, Junhao Mei, Huanglin Dong, Shaobing Peng, Jianliang Huang, Kehui Cui, Lixiao Nie

**Affiliations:** 1 National Key Laboratory of Crop Genetic Improvement, MOA Key Laboratory of Crop Ecophysiology and Farming System in the Middle Reaches of the Yangtze River, College of Plant Science and Technology, Huazhong Agricultural University, Wuhan, Hubei 430070, China; 2 Hubei Collaborative Innovation Center for Grain Industry, Yangtze University, Jingzhou, Hubei 434023, China

## Abstract

Double direct-seeding for double rice cropping is a simplified, labor saving, and efficient cropping system to improve multiple-crop index and total rice production in central China. However, poor crop establishment of direct-seeded early rice due to chilling stress is the main obstacle to wide spread of this system. A series of experiments were conducted to unravel the effects of pre-sowing seed treatments on emergence, seedling growth and associated metabolic events of direct-seeded early rice under chilling stress. Two seed priming treatments and two seed coating treatments were used in all the experiments. A non-treated control treatment was also maintained for comparison. In both the field and growth chamber studies, seed priming with selenium or salicylic acid significantly enhanced the emergence and seedling growth of rice compared with non-treated control. Nevertheless, such positive effects were not apparent for seed coating treatments. Better emergence and vigorous seedling growth of rice after seed priming was associated with enhanced α-amylase activity, higher soluble sugars contents, and greater respiration rate in primed rice seedlings under chilling stress. Taking together, these findings may provide new avenues for understanding and advancing priming-induced chilling tolerance in direct-seeded early rice in double rice cropping system.

Double rice cropping is a typical rice production system in central China and is considered as an efficient system to improve multiple-crop index and total rice production^1^. In the traditional double rice cropping system in China, seeds are sown in nursery bed and seedlings are pulled out and transplanted in fields at 2–4 weeks after sowing. However, planting area of the traditional double rice cropping system in China continued to decline because of the labor shortage in county and relatively low production efficiency for this cropping system^2^.

Replacing transplanting by direct-seeding may be an option to sustain rice production in double rice cropping area. Double direct-seeding for double rice cropping is a simplified, labor saving, and efficient cropping system in central China. It refers to the process of crop establishment by directly sowing the dry or soaked seeds in the field in both early and late seasons rather than nursery transplanting. Without sprout cultivation and transplanting, double direct-seeded rice consumed less irrigation water and demands less labor, thus reducing the total inputs^3^.

Since 1970s, some researches have been conducted to probe the suitable rice varieties, crop management, and weed control in double direct-seeded rice system in central China^4^,^5^. However, poor crop establishment of direct-seeded early rice due to chilling stress is the main obstacle to wide spread of this system. In order to ensure the rice production of double direct-seeded rice, early rice must be directly sown in early or middle of April in central China. It has been reported that the daily mean temperature in central China during April is 16.4 °C and the cold spell (the duration of low temperature that is below 10 °C lasts for more than three days) frequently occurs in this area. In some years, the daily mean temperature even dropped to 7.1 °C^6^.The low temperature is known to severely hamper the seed germination and seedling growth of the direct-seeded early rice. In 2008 and 2010, seedling emergence rates of the direct-seeded early rice were decreased by 38-55% due to low temperature and the severe cold spell in central china^7^. Therefore, sustainable and effective technologies are inevitable to improve the seed germination and enhance the seedling growth of the early direct-seeded rice under chilling stress.

Pre-sowing seed treatment such as seed coating and seed priming could improve the seed germination and seedling vigor particularly under unfavorable environmental conditions. Seed coating is one of the pre-sowing seed treatments that have widely applied for many crops around the world^8^. The coating agents are generally composed of pesticide, bactericide, micro-fertilizer and plant growth regulator^9^ and it has been proved that seed coating could increase germination, enhance seedling growth, and reduce pests and diseases^10^. Hanyubaomu (HYBM) and Miaoboshi (MBS) are the two typical rice coating agents, which are proposed to improve stress tolerance during seed germination and promote seedling growth under chilling and saline stress^11^,^12^. Seed priming is a controlled hydration technique that allows the pre-germination metabolisms without actual germination^13^. It is one of the most effective, pragmatic and short-term approaches for increasing seed vigor, synchronization of germination under different stresses^14^. Primed seeds could exhibit faster and uniform emergence and better crop establishment by providing a vigorous head start^15^. Recently, Khaliq *et al*. has reported that selenium priming could trigger seedling growth and early commencement of emergence in rice^16^. Farooq *et al*. found that seed priming with salicylic acid was effective in improving the emergence and seedling growth of maize seedlings under chilling stress^17^. Likewise, Pouramir *et al*. reported that salicylic acid priming enhanced the activity of antioxidant enzymes in rice seedlings under chilling stress^18^.

Starch metabolism has been considered to play a key role in early seedling vigor. Williams and Peterson have reported that the α-amylase activity and total soluble sugar contents in the rice seeds were strongly linked with the seed germination and seedling growth of rice^19^. Results of Lee and Kim depicted that seed priming was helpful in enhancing the starch metabolism in the rice seeds^20^. Likewise, the rise in respiration rate has been recognized as a signal of seed germination, and the high respiration rate was strongly correlated with the rapid seed germination^21^. Therefore, the activity of starch metabolism and the respiration rate should be the important traits for reflecting the seedling vigor particularly under stress conditions.

Previously, the effectiveness of pre-sowing seed treatment on emergence and seedling growth of direct-seeded early rice under chilling stress has rarely been studied in central China. Therefore, the objectives of this study were (1) to examine the effects of seed coating and priming treatments on seed germination and seedling growth of direct-seeded early rice under chilling stress, (2) to explore the biochemical and physiological changes in rice seedlings induced by seed coating and priming treatments in response to chilling stress, and (3) to identify the most suitable “pre-sowing seed treatment” for direct-seeded early rice in central China.

## Results

### Field experiment: Germination and seedling growth of direct-seeded early rice under different pre-sowing seed treatments

#### Soil temperature

**Figure 1:**
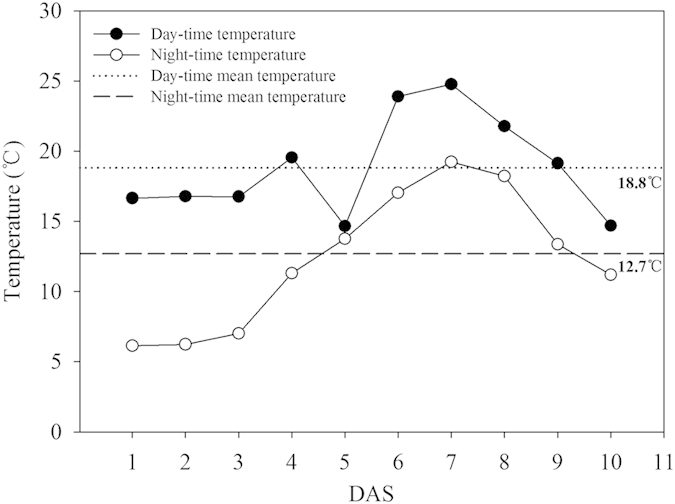
Day-time, night-time, day-time mean, and night-time mean soil temperatures during seed germination (1–10 DAS) of direct-seeded early rice in a field experiment. DAS: days after sowing. The upper straight line represented the day-time mean temperature (18.8 °C) during seed germination; the lower straight line represented the night-time mean temperature (12.7 °C) during seed germination.

The data regarding mean soil temperature during 1–10 days after sowing (DAS) were shown in Fig. 1. During 1–10 DAS, the daily mean temperature, mean day-time temperature, and mean night-time temperature were 16.0 °C, 18.8 °C, and 12.7 °C, respectively.

#### Rice emergence

**Figure 2:**
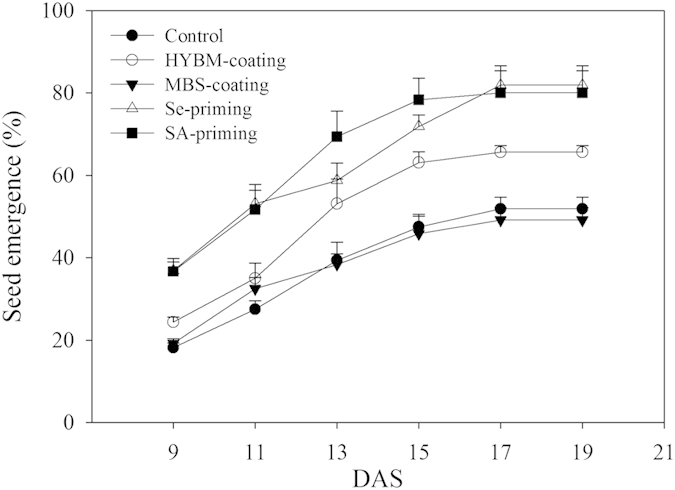
Emergence dynamics of HYBM-coated, MBS-coated, Se-primed, SA-primed, and non-treated seeds of direct-seeded early rice in a field experiment. (DAS: days after sowing, HYBM-coating: Hanyubaomu coating, MBS-coating: Miaoboshi coating, the ratio of seed weight to coating agent weight was 8:1 for HYBM, and 40:1 for MBS; Se-priming: priming with 50 M sodium selenite, SA-priming: priming with 100 mg/L salicylic acid). Rice cultivar Huanghuazhan (HHZ) was used in field experiment. The seed emergence was slow because of the chilling stress, therefore, the seed emergence data were recorded from 9 DAS till constant at 19 DAS. Error bars indicate standard error (n = 4).

Significant variations in emergence of rice were observed under the influence of chilling stress and different pre-sowing seed treatments (Fig. 2). Under chilling stress, the both seed priming treatments and HYBM-coating treatment significantly enhanced the emergence of rice compared with non-treated control, nevertheless, MBS-coating was not effective for rice emergence. In non-treated control, the rice emergence rate was 18.13% at 9 DAS, and the final emergence was only 51.87% at 19 DAS. At 19 DAS, the maximum emergence was observed for selenium (Se) priming (81.87%), followed by salicylic acid (SA) priming treatment (80.00%). In HYBM-coating and MBS-coating treatments, only 65.62% and 49.16% seeds could germinate at 19 DAS. The MBS-coating was statistically similar with non-treated control regarding final emergence of rice.

#### Seedling growth

Seed-priming treatments significantly enhanced the seedling growth of rice compared with non-treated control, nevertheless, the effect of seed-coating treatments was limited under chilling stress (Table 1). On average, seed priming treatments significantly increased the root length, shoot length, root fresh weight and shoot fresh weight by 27.77%, 18.47%, 31.73% and 26.24%, respectively compared with control. The respective enhancements for seed coating treatments were 16.57%, 8.28%, 10.96% and 11.30% in comparison with control. Both seed coating treatments were statistically similar with non-treated control for all seedling growth attributes except for the root fresh weight in HYBM-coating treatment (Table 1). No significant difference was observed between Se-priming treatment and SA-priming treatment, as well as between HYBM-coating treatment and MBS-coating treatment.

### Growth chamber experiment: Germination, seedling growth and biochemical attributes of rice under different pre-sowing seed treatments and chilling stress

#### Germination

**Figure 3:**
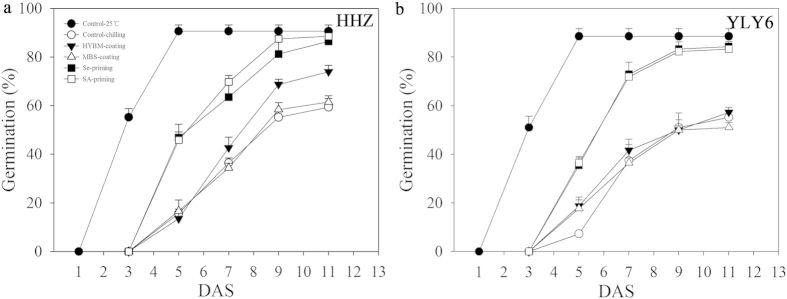
Germination dynamics of HYBM-coated, MBS-coated, Se-primed, SA-primed, and non-treated seeds of rice in growth chamber experiment-1. (**a**) HHZ. (**b**) YLY6. (HHZ: inbred variety huanghuazhan, YLY6: hybrid variety Yangliangyou-6, Control−25 °C: normal temperature control using non-treated seeds, Control-chilling: low temperature control using non-treated seeds, DAS: days after sowing, HYBM-coating: Hanyubaomu coating, MBS-coating: Miaoboshi coating, the ratio of seed weight to coating agent weight was 8:1 for HYBM and 40:1 for MBS; Se-priming: priming with 50 μM sodium selenite, SA-priming: priming with 100 mg/L salicylic acid). The seed germination data were recorded from 5 DAS till constant at 11 DAS. Error bars indicate standard error (n = 4).

Based on the average day-time and night-time temperatures during the 1–10 DAS in the field experiment, the low temperature treatment for the growth chamber experiment was maintained at 18.8 °C during the day-time, and 12.7 °C during the night-time (Fig. 1). The temperature for non-stressed control (normal temperature) was set at 25 °C for 24h constantly. Chilling stress was found to severely reduce the germination of the rice seeds under growth chamber conditions as compared with normal temperature. The final germination percentages of non-treated seeds of HHZ and YLY6 under chilling stress were reduced by 52.63% and 60.38%, respectively as compared with normal temperature control. However, the Se-priming and SA-priming treatments significantly enhanced the germination of both rice cultivars under chilling stress. On average, the seed priming treatments significantly increased the germination of HHZ and YLY6 by 87.50% and 82.81%, respectively as compared with low temperature control and the final seed germination of the priming treatments was almost similar with that under normal temperature control. The HYBM-coating treatment was found to only enhance the germination of HHZ by 73.96%, while the MBS-coating treatment was ineffective for both rice cultivars regarding seed germination (Fig. 3). No obvious difference in seed germination was found between the varieties and seed priming treatments.

#### Seedling growth

Chilling stress severely hampered the seedling growth of both rice cultivars compared with the normal temperature control. Nevertheless, seed priming treatments depicted significant (p ≤ 0.05) improvement in early seedling growth of both rice cultivars under chilling stress, The seed coating treatments did not significantly influence the seedling growth of both cultivars, except for the shoot length of YLY6 in MBS-coating. Both the cultivars showed similar response to pre-sowing seed treatments (Table 2). In HHZ, seed priming treatments averagely increased the root length, shoot length, root fresh weight and shoot fresh weight of rice by 57.75%, 72.54%, 116.18%, and 118.11%, respectively, compared with non-treated control under chilling stress. The respective increments for YLY6 were 71.5%, 97.90%, 91.95%, and 108.28%, respectively (Table 2). The variations between Se-priming and SA-priming treatments regarding seedling growth parameters were not significant (except for the root weight in HHZ), as well as between the two seed coating treatments.

#### Starch metabolism

**Figure 4:**
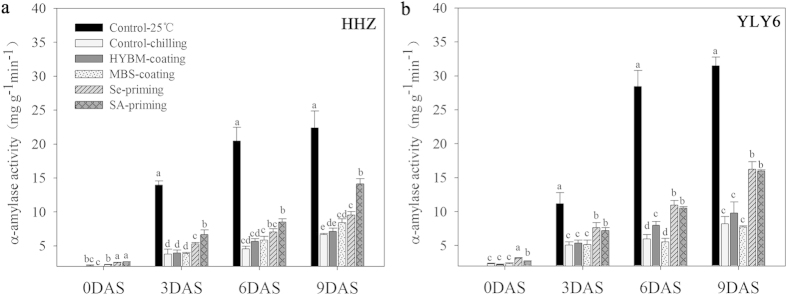
Variations in α-amylase activity of rice seeds and seedlings in HYBM-coated, MBS-coated, Se-primed, SA-primed, and non-treated seed treatments at 0, 3, 6, and 9 DAS in growth chamber experiment-1. (**a**) HHZ. (**b**) YLY6. (HHZ: inbred variety huanghuazhan, YLY6: hybrid variety Yangliangyou-6, Control−25 °C: normal temperature control using non-treated seeds, Control-chilling: low temperature control using non-treated seeds, DAS: days after sowing, HYBM-coating: Hanyubaomu coating, MBS-coating: Miaoboshi coating, the ratio of seed weight to coating agent weight was 8:1 for HYBM, and 40:1 for MBS; Se-priming: priming with 50 μM sodium selenite, SA-priming: priming with 100 mg/L salicylic acid). Seeds at 0 DAS were tested under dry weight basis, while seedlings at 3, 6, and 9 DAS were tested under fresh weight basis. Different lowercase letters denote statistical differences among treatments of a cultivar at the 5% level according to LSD test. Error bars above mean indicate standard error (n = 4).

**Figure 5:**
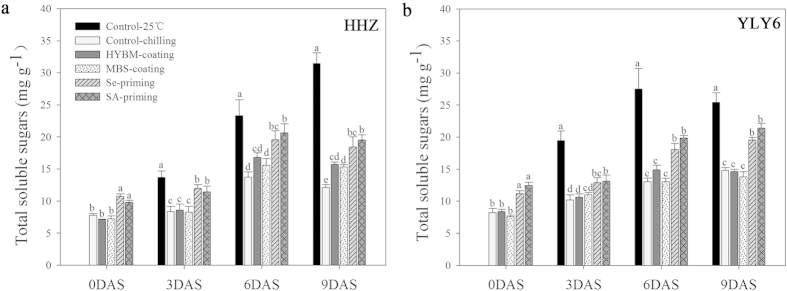
Variations in total soluble sugar content of rice seeds and seedlings in HYBM-coated, MBS-coated, Se-primed, SA-primed, and non-treated seed treatments at 0, 3, 6, and 9 DAS in growth chamber experiment-1. (**a**) HHZ. (**b**) YLY6. (HHZ: inbred variety huanghuazhan, YLY6: hybrid variety Yangliangyou-6, Control−25 °C: normal temperature control using non-treated seeds, Control-chilling: low temperature control using non-treated seeds, DAS: days after sowing, HYBM-coating: Hanyubaomu coating, MBS-coating: Miaoboshi coating, the ratio of seed weight to coating agent weight was 8:1 for HYBM, and 40:1 for MBS; Se-priming: priming with 50 μM sodium selenite, SA-priming: priming with 100 mg/L salicylic acid). Seeds at 0 DAS were tested under dry weight basis, while seedlings at 3, 6, and 9 DAS were tested under fresh weight basis. Different lowercase letters denote statistical differences among treatments of a cultivar at the 5% level according to LSD test. Error bars above mean indicate standard error (n = 4).

Starch metabolism in rice seedlings was assessed in terms of α-amylase activity and total soluble sugar content (Figs 4 and 5). Chilling stress reduced the activity of α-amylase activity and total soluble sugar content in both rice cultivars as compared with normal temperature control. Under chilling stress, both the Se-priming and SA-priming treatments significantly increased the starch metabolism of dry seeds as well as rice seedlings, while seed coating treatments were statistically similar with non-treated control. In this study, α-amylase activity and total soluble sugar content were recorded at 0, 3, 6, 9 DAS. In the two seed priming treatments, both α-amylase activity and total soluble sugar content were progressively increased even from 0 DAS and achieved the highest values at 9 DAS (except that the soluble sugar content in HHZ were maximum at 6 DAS). However, such positive effects were not found in the both seed coating treatments. When averaged across two rice cultivars, both seed priming treatments averagely increased the α-amylase activity and total soluble sugar contents by 93.56% and 46.61%, respectively at 9 DAS in comparison with low temperature control. While on average, both seed coating treatments increased the α-amylase activity and total soluble sugar by only 11.96% and 12.04%, respectively. In HHZ, SA-priming treatment recorded significantly higher α-amylase activity than that in Se-priming treatment at 3 and 9 DAS, while these two treatments were statistically similar with each other in YLY6 regarding α-amylase activity and total soluble sugar content.

#### Respiration rate

**Figure 6:**
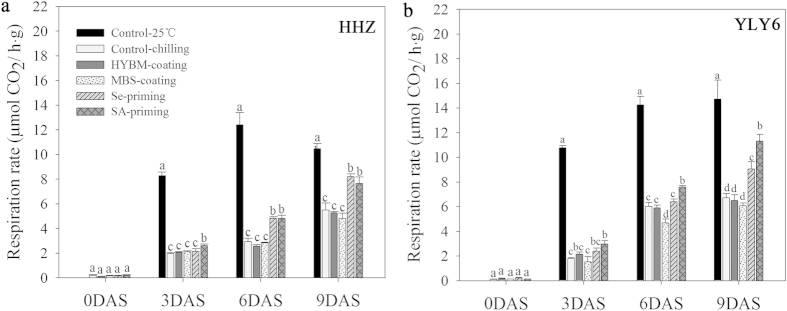
Variations in respiration rate of rice seeds and seedlings in HYBM-coated, MBS-coated, Se-primed, SA-primed, and no-primed and no-coated seed treatments at 0, 3, 6, and 9 DAS in growth chamber experiment-2. (**a**) HHZ. (**b**) YLY6. (HHZ: inbred variety huanghuazhan, YLY6: hybrid variety Yangliangyou-6, Control−25 °C: normal temperature control, Control-chilling: low temperature control, DAS: days after sowing, HYBM-coating: Hanyubaomu coating, MBS-coating: Miaoboshi coating, the ratio of seed weight to coating agent weight was 8:1 for HYBM and 40:1 for MBS; Se-priming: priming with 50 μM Sodium selenite, SA-priming: priming with 100 mg/L Salicylic acid). Seeds at 0 DAS were tested under dry weight base, while seedlings at 3, 6, and 9 DAS were tested under fresh weight base. Different lowercase letters denote statistical differences between treatments of a cultivar at the 5% level according to LSD test. Error bars above mean indicate standard error (n = 4).

Chilling stress had a negative effect on respiration rate of the rice seedlings in both cultivars. However, seed priming treatments were effective to significantly enhance the respiration rate under chilling stress. None of the seed coating treatment could significantly increase the respiration rate during rice germination as compared with non-treated control (Fig. 6). At 0 and 3 DAS, no variance was observed among the pre-sowing seed treatments in both cultivars, except for relatively higher respiration rate in the SA-priming treatment at 3 DAS. At 6 and 9 DAS, the respiration rate in both seed priming treatments showed a prominent and significant increase compared with non-treated control, while the respiration rate did not increase in both seed coating treatments (Fig. 6). At 9 DAS, the respiration rates of Se-primed and SA-primed rice seedlings were increased by 40.10% and 47.81%, respectively, when averaged across cultivars. While a significant decrease in respiration rate was observed in the MBS-coating treatment in YLY6 at 6 DAS.

## Discussion

Chilling stress is one of the serious environmental stresses affecting growth and development of rice. In our field study, the daily mean temperature and the minimum mean temperature were 16.0 °C and 12.7 °C, respectively. Previously, Yoshida indicated that the speed and percentage of rice germination can be decreased under daily mean temperature of below 20 °C^22^. Moreover, Sipaseuth *et al*. reported that the establishment of rice seedling was severely hampered, when the mean minimum temperature was below 16 °C^23^.These studies suggested that the direct-seeded early rice was exposed to chilling stress during the seed germination in our studie**s**.

In our field experiment, the emergence of rice in non-treated control treatment was extremely low (Figs 2 and 3). Under optimum temperature, it has been reported that the rice seed germination usually reached 90% at 3 DAS^22^, which indicated that the speed and percentage of rice emergence were drastically reduced by chilling stress in our field experiment. In growth chamber experiment, chilling stress also negatively affected the germination and seedling growth of rice as compared with normal temperature control (Tables 1 and 2; Figs 2 and 3). Our results were consistent with the previous researches that reported the delayed germination and poor and non-uniform seedling growth under chilling stress^24^,^25^. Li *et al*. reported that low temperature hindered the water uptake and root growth, resulting in a decreased seed germination^26^. Chilling stress during seed imbibition phase may lead to increasing escape of solutes from the seeds, such as amino acids and carbohydrates due to the destruction of membrane integrity in rice seeds^27^. The reduction in coleoptile growth during germination process may be attributed to the direct effect of chilling stress on cell elongation and division, or indirectly due to metabolic imbalance^28^.

Present study examined the effect of pre-sowing seed treatments on seed germination and seedling growth in direct-seeded early rice under low temperature conditions. The results demonstrated that both seed priming treatments efficiently improved the emergence and seedling growth performance under chilling stress while such positive effects were not apparent for seed coating treatments (Tables 1 and 2; Figs 2 and 3). Better ability of primed seeds to complete the germination process in a short time and cope with low temperature conditions might be attributed to readily available substance for germinating seedlings. Pereira *et al*. evaluated the effect of seed priming in carrot under low temperature conditions and reported the improved field emergence rate by seed priming^29^. Wahid *et al*. stated that seed priming enhanced the germination rate, speed and uniformity under multiple abiotic stress^30^. In present study, seed coating treatments slightly enhanced the germination and seedling growth under low temperature conditions, but the effects were limited and were variable to different cultivars (Tables 1 and 2; Figs 2 and 3). The HYBM-coating treatment was effective in enhancing the germination of HHZ, while seed germination and seedling growth of both rice cultivars in MBS-coating treatment were similar to non-treated control under chilling stress. Previously, Xiao reported that MBS-coating in rice increased the emergence rate by 6–16%^31^. Moreover, Zeng and Shi reported that seed coating could stimulate the emergence of rice seedlings and increase its root activity^32^. However, some researches have reported the negative effects of seed coating on seed germination^33^,^34^, suggesting that the effects of seed coating were not consistent. The HYBM coating agents used in present study were composed of carbendazim, paclobutrazol and mineral clay, and the components of MBS coating agents were prochloraz, imidacloprid, paclobutrazol and polymer materials. The effect of the seed-coating treatments on germination might be diluted under chilling stress due to the complicated component of the coating agents, as the polymer materials were impermeable, which would have reduced the gas and water exchange from soil to the seeds^35^. The coating agent may be separated from the seeds after raining, and it has also be found that the bactericide might hamper the germination of the seed at low temperature^36^. The stability of the seed-coating agents and the factors that influence the effects of seed coating under chilling stress still needs be study further.

Our results depicted that Se-priming and SA-priming treatments were effective in enhancing seed germination and seedling growth of direct-seeded early rice under chilling stress. The positive effects induced by Se-priming treatment might be due to the modulation of the sugar flux that might act as a molecular signal for the activation of the plant response^37^,^38^. While salicylic acid is a plant hormone and is recognized as an endogenous signal, mediating in plant defense system in particular against pathogens^39^. It has been known to provide protection for cell structure^40^, conceal the reactive oxygen species (ROS) production and enhance the activity of antioxidants^41^.

In our present study, pronounced increases in α-amylase activity and total soluble sugar contents were recorded in seed priming treatments under chilling stress (Figs 4 and 5). Higher α-amylase activity and total soluble sugar contents in primed seeds and seedlings were associated with the better seed germination/emergence and faster seedling growth. Correlation analysis showed that starch metabolism and respiration rate were significantly correlated with seed germination, and seedling growth attributes of rice (Table 3), which is in consistent with the previous studies that germination and seedling growth were strongly linked with starch metabolism both in rice seeds and its seedlings^19^,^20^,^21^. Seed priming treatments significantly increased the α-amylase activity (on average 87.37%, 9 DAS) and total soluble sugars (on average 46.61%, 9 DAS) in both rice cultivars (Figs 4 and 5), possibly because starch degradation was activated and seed reserves were highly enhanced by seed priming treatments under chilling stress. The ability of plants to degrade starch into soluble sugars probably plays a key role in their ability to survive and grow faster under wide range of environments. In rice, amylase activity is highly induced during germination^42^. Higher α-amylase activity in primed seeds and seedlings is also reflected through higher soluble sugar concentrations and faster rate of starch breakdown in germinating primed seeds, which presumably provided the substrates necessary for generating the energy required for growth and maintenance processes^15^.

Higher starch metabolism in rice after seed priming might also be attributed to higher respiration rate (Fig. 6), through which more ATP might be generated to accelerate the growth of radicle and coleoptile. Palmiano and Juliano suggested the rapid increase of respiration rate was coincided with the emergence of radicle^43^. Paul and Mukherji reported that the rise in respiration rate was due to the activation of α-amylase enzymes^44^, which indicated that the increase in respiration rate of primed seedlings was strongly linked with the activated starch metabolism and the subsequent growth of radicles. The slight effect of seed coating treatments on enhancement of germination and seedling growth were also associated with the limited increase in starch metabolism, as well as respiration rate.

Under chilling stress, the responses of seed germination and seedling growth to pre-sowing seed treatments in growth chamber experiment were consistent with that in the field experiment. Moreover, the responses of two rice cultivars to different seed pre-sowing treatments were similar and consistent, suggesting the effectiveness of treatments across cultivars and experiments.

In crux, present studies demonstrated that seed priming with Se or SA was effective to significantly enhance the emergence and seedling growth performance of direct-seeded early rice under chilling stress compared with non-treated control. Nevertheless, such positive effects were not apparent for seed coating treatments. Better emergence and vigorous seedling growth of rice after seed priming was associated with enhanced α-amylase activity, higher soluble sugars contents, and greater respiration rate under chilling stress. However, further transcriptomic/proteomic studies are needed to explore the molecular mechanisms of seed priming-induced chilling tolerance.

## Materials and Methods

### Seed source

Seeds of two widely grown indica rice (*Oryza sativa* L.) cultivars viz., Huanghuazhan (HHZ, inbred) and Yangliangyou-6 (YLY6, hybrid) were obtained from Crop Physiology and Production Center, Huazhong Agricultural University, Wuhan, China. Both cultivars have the initial germination of >95%. The initial seed moisture content was below 10.0% (on dry weight basis). Under field conditions, only a single cultivar (HHZ) was used, while in growth chamber experiment, an additional cultivar (YLY6) was used in order to examine the genotypic variations in response to seed treatments. All the seeds used in this study were selected from the same lot.

### Seed coating treatment

Two widely used commercial rice coating agents, Hanyubaomu (HYBM) and Miaoboshi (MBS) were used in this study. The ratio of seed to HYBM was 8:1, while seed to MBS was 40:1 (recommended by the producer) on dry weight basis. For HYBM-coating treatment, the seeds were soaked in distilled water for 20 minutes and then the excess water was blot up using bilbulous paper (blot paper). Then the coating agents and seeds were put in a circular bottom container, where the seeds and the coating agents were stirred up until the agents were evenly wrapped on the seeds^45^. For MBS-coating treatment, the coating agent was mixed with distilled water, the ratio of coating agent weight to distilled water volume (w/v) was 1:1. Dry seeds and the coating agents/water mixture were mixed in a circular bottom container until the agents were evenly wrapped on the seeds. After seed coating, the seeds were transferred to air dry oven at 25 °C for 48 h to reduce the moisture contents to <10%. After drying, seeds were sealed in plastic bags then stored in refrigerator (–4 °C). The seed coating were done five days prior to sowing.

### Seed priming treatment

Seed priming treatments were pre-optimized in various preliminary studies. Seed priming treatments selected for this study were: Se (50 μM sodium selenite), SA (100 mg/L salicylic acid). Seeds were primed in dark at 25 °C for 24 h, with constant gentle agitation. The ratio of seed weight to solution volume (w/v) was 1:5 and priming solution was changed after every 12 h. After 24 h, the primed seeds were washed with distilled water for 2 mins, surface dried and transferred to air dried oven at 25 °C for 48 h to reduce the moisture contents to <10%. After drying, seeds were sealed in plastic bags then stored in refrigerator (−4 °C). Like seed coating, seed priming was also done five days prior to sowing.

## Experimentation

### Field experiment

The field experiment was carried out at the experimental station of Zhougan Village, Dajin Town, Wuxue Country, Hubei Province, China (29°51′N 115°33′E). The treatments comprised of a non-treated control, hanyubaomu coating (HYBM-coating), miaoboshi coating (MBS-coating), selenium priming (Se-priming) and salicylic acid priming (SA-priming). Prior to sowing, the field was flooded and puddled, followed by draining of excess water. The experiment was arranged in a randomized complete block design with four replications. The net plot size for each treatment was 4 m^2^ (2 m × 2 m), and 9 rows were sown in each plot. Rice seeds were manually sown at 20 cm row to row and 5 cm plant to plant distance on 10^th^ April 2015. The soil was kept saturated throughout the experiment. After sowing, the soil temperature was continuously recorded using HOBO data logger with 3 detectors, which were placed into the soil depth of 5 cm.

Emergence of seeds was recorded on daily basis according to AOSA^46^ and was expressed as percentage. At 12 DAS, two lines (rows) from the center of the plot were chosen and 10 seedlings from each line were sampled for the determination of seedling growth. After measuring shoot length and the maximum root length, all the seedlings were dissected into root and shoot for determination of their fresh weights.

## Growth chamber experiment

### Experiment-1

In order to examine the biochemical and physiological changes after seed coating and seed priming treatments, a growth chamber experiment was carried out in Crop Physiology and Production Center, Huazhong Agricultural University, Wuhan, China. The plastic trays with 31.5 cm × 20.0 cm × 12.0 m size were filled with 5 kg of air-dried soil which was collected from the field, where field experiment was conducted. The soil was flooded for 12h, then the excess water was drained and soil surface was leveled. The pre-sowing seed treatments were the same as used in the field study. Two cultivars viz., HHZ and YLY6 were used in this study in order to verify the intrinsic changes of the rice induced by pre-sowing seed treatment were similar for different cultivars.

In each tray, 10 rows (two rows for each treatment) of rice seeds were equally sown on the soil surface (12 seeds for each row). The experiment was laid out in a completely randomized design with factorial arrangement replicated 8 times. Four replications of trays were used to record seed germination and seedling growth parameters. While the seedlings in the other four replications, were sampled for determinations of α-amylase activity and soluble sugar contents. The soil in the trays was kept saturated to ensure enough water for seed germination. All the trays were placed in growth chamber with 12 h light period, 18.8 °C day and 12.7 °C night temperatures according to the mean day-time and night-time temperatures recorded during 1–10 DAS in the field study. In addition, a normal temperature control treatment was set using a separate growth chamber, where the temperature was kept constant at 25 °C.The humidity during the course of study was maintained at 60% in both growth chambers. Equal volume of distilled water was applied to all trays when their moisture content declined.

Germination of seeds was recorded on daily basis according to AOSA until a constant count was achieved^46^. Seeds were considered to be germinated when hypocotyl length exceeded 2 mm. Germination percentage was taken as the ratio of number of seeds germinated to the total number of seeds sown and was expressed as percentage. At 12 DAS, 10 seedlings were randomly sampled from each treatment (4 replicates) to record their shoot and root length. The seedlings were dissected into roots and shoots and their fresh weights were recorded immediately.

For determination of α-amylase activity, 1.0 g dry seeds (0 DAS) and seedling samples at 3, 6 and 9 DAS including shoot and root were ground and mixed with 100 ml distilled water, and left for 24 h at 4 °C then filtered the mixture with Whatman No. 42. The enzyme activity was determined by dinitro-salicyclic acid (DNS) method^47^. In order to determine total soluble sugar contents, 0.5 g dry seeds (0 DAS) and the seedling samples at 3, 6 and 9 DAS were ground and then mixed with 50 ml distilled water, and left for 24 h at 25 °C^20^. Mixture was filtered, and the total soluble sugar contents were determined by the phenol sulfuric method^48^.

### Experiment-2

In order to examine the seed respiration rate induced by pre-sowing treatments, 25 g of seeds from each treatment were evenly germinated on two layers of filter paper in 14.5 cm diameter Petri dishes. After adding 20 ml water to each replicate, Petri dishes were covered with lid and placed on steel racks in a growth chamber. The cultivars, pre-sowing seed treatments, and the temperature settings were the same to experiment-1. The humidity during the course of study was maintained at 60%. Equal volume of distilled water was applied to all Petri dishes when their moisture content declined. All the treatments were arranged in a completely randomized design under factorial arrangement with four replications. Five grams of dry seeds or seedlings from each replicate were sampled and weighted at 0, 3, 6, and 9 DAS, then their respiration rate was determined immediately using small-skep-method^49^. Briefly, 5 g of seeds or seedlings were put into a 0.5 L of glass bottle, and the bottle was connected to a close-circuit system, the CO_2_ concentration in this bottle was record after every minute. Respiration rate was calculated based on the increase in CO_2_ concentrations within one minute.

## Statistical analysis

All data from the field and growth chamber experiments are presented as the mean value ± standard error (SE) of four replicates. Analysis of variance was performed using Statistix 9.0. The differences among treatments were separated using Least Significance Difference (LSD) test at 0.05 probability level.

## Additional Information

**How to cite this article**: Wang, W. *et al*. Pre-sowing Seed Treatments in Direct-seeded Early Rice: Consequences for Emergence, Seedling Growth and Associated Metabolic Events under Chilling Stress. *Sci. Rep.* **6**, 19637; doi: 10.1038/srep19637 (2016).

## Figures and Tables

**Table 1:** The effect of different pre-sowing seed treatments on seedling growth attributes of direct-seeded early rice at 12 DAS in a field experiment.

Variety	Pre-sowing seed treatment	Root length	Shoot length	Root FW	Shoot FW
(cm)	(cm)	(mg seedling^−1^)	(mg seedling^−1^)		
Huanghuazhan	Control	3.41±0.17 b	3.32±0.12 c	9.03±0.67 d	15.97±0.67 c
	HYBM-coating	3.93±0.23 ab	3.55±0.07 bc	10.59±0.65 bc	17.50±0.78 bc
	MBS-coating	4.02±0.29 ab	3.64±0.27 abc	9.45±0.65 cd	18.05±0.58 bc
	Se-priming	4.30±0.21 a	4.02±0.14 a	11.28±0.41 ab	20.97±0.91 a
	SA-priming	4.42±0.50 a	3.85±0.16 ab	12.51±0.30 a	19.34±1.13 ab

^†^Different lowercase letters denote statistical differences between treatments at the 5% level according to LSD test. DAS: days after sowing, HYBM-coating: Hanyubaomu coating, MBS-coating: Miaoboshi coating, the ratio of seed weight to coating agent weight was 8:1 for HYBM and 40:1 for MBS; Se-priming: priming with 50 μM Sodium selenite, SA-priming: priming with 100 mg/L Salicylic acid.

**Table 2:** Seedling growth attributes of two rice cultivars under the influence of pre-sowing seed treatments and chilling stress at 12 DAS in growth chamber experiment-1.

Variety	Pre-sowing seed treatment	Root length	Shoot length	Root FW	Shoot FW
		(cm)	(cm)	(mg seedling^−1^)	(mg seedling^−1^)
Huanghuazhan	Control−25 °C	9.94 ± 0.63a	25.29 ± 0.65a	26.91 ± 3.29 a	51.94 ± 1.12 a
	Control-chilling	2.00 ± 0.32 c	1.22 ± 0.28 c	2.75 ± 0.43 d	3.23 ± 0.62 c
	HYBM-coating	2.25 ± 0.22 c	1.19 ± 0.20 c	3.49 ± 0.46 d	2.81 ± 0.62 c
	MBS-coating	2.23 ± 0.20 c	1.25 ± 0.19 c	3.03 ± 0.28 d	3.08 ± 0.77 c
	Se-priming	3.11 ± 0.37 b	1.99 ± 0.37 b	5.21 ± 0.52 c	7.57 ± 0.55 b
	SA-priming	3.20 ± 0.30 b	2.22 ± 0.36 b	6.68 ± 0.92 b	6.52 ± 0.94 b
					
Yangliangyou-6	Control−25 °C	8.38 ± 0.54 a	25.91 ± 1.16 a	27.70 ± 1.43 a	68.66 ± 3.01 a
	Control-chilling	2.00 ± 0.13 c	1.59 ± 0.21 d	5.78 ± 0.63 c	7.48 ± 0.54 c
	HYBM-coating	2.06 ± 0.07 c	1.74 ± 0.17 cd	6.82 ± 0.89 c	7.37 ± 0.95 c
	MBS-coating	2.32 ± 0.29 c	2.04 ± 0.09 c	6.88 ± 0.36 c	7.49 ± 1.15 c
	Se-priming	3.65 ± 0.26 b	3.60 ± 0.07 b	9.63 ± 0.12 b	14.71 ± 0.26 b
	SA-priming	3.76 ± 0.11 b	3.50 ± 0.16 b	9.76 ± 0.47 b	14.98 ± 0.72 b

^†^Different lowercase letters denote statistical differences between treatments of a cultivar at the 5% level according to LSD test. DAS: days after sowing, Control−25 °C: normal temperature control using non-treated seeds, Control-chilling: low temperature control using non-treated seeds. HYBM-coating: Hanyubaomu coating, MBS-coating: Miaoboshi coating, the ratio of seed weight to coating agent weight was 8:1 for HYBM and 40:1 for MBS; Se-priming: priming with 50 μM sodium selenite, SA-priming: priming with 100 mg/L salicylic acid.

**Table 3:** Correlation analysis of α-amylase activity, total soluble sugars, and respiration rate with rice germination percentage and seedling growth attributes in growth chamber experiments.

	α-amylase activity	Total soluble sugars	Respiration rate
Root length	0.9077**	0.9339**	0.8884**
Shoot length	0.9197**	0.7834**	0.9084**
Root fresh weight	0.8479**	0.6798*	0.8443**
Shoot fresh weight	0.8465**	0.7048*	0.9102**
Germination percentage	0.8054**	0.6439**	0.7577**

^†^The values refer to the correlation coefficients. *denotes statistical correlation between treatments at the 5% level. **denotes statistical differences between treatments at the 1% level according to t test.
